# Isolated oropharyngeal abscess with hypopharyngeal extension recurring 12 years after initial surgical management: A case report and review of the literature

**DOI:** 10.1177/2050313X221089119

**Published:** 2022-04-04

**Authors:** Michel Khoury, Selina Xiangxu Dong, Hussain Alsaffar, Stephanie Johnson-Obaseki, Lisa Caulley

**Affiliations:** 1 Department of Undergraduate Medical Education, Faculty of Medicine, University of Ottawa, Ottawa, ON, Canada; 2 Department of Otolaryngology—Head and Neck Surgery, The Ottawa Hospital, University of Ottawa, Ottawa, ON, Canada; 3 Department of Epidemiology, Erasmus MC, Rotterdam, The Netherlands; 4 Knowledge Synthesis Group, Clinical Epidemiology Program, The Ottawa Hospital Research Institute, Ottawa, ON, Canada

**Keywords:** Hypopharynx, oropharynx, abscess, deep neck infection, case report, review of literature

## Abstract

Pharyngeal abscesses require urgent management as they have the propensity to cause severe and life-threatening complications. The introduction of antibiotics has led to a dramatic decline in the incidence of these infections. Regardless, abscess formation continues to be observed in the peritonsillar, parapharyngeal, and retropharyngeal spaces. Oropharyngeal and hypopharyngeal abscesses that cause airway obstruction are scarcely reported and tend to be secondary to other processes. Herein, we describe the case of an 83-year-old man presenting with an idiopathic, obstructive, oropharyngeal wall abscess, extending from the infratonsillar region to the hypopharynx, which recurred after initial surgical management 12 years prior for the same process. He required reintervention during both episodes for rapid reaccumulation. A detailed electronic literature search of PubMed and MedLine was performed for studies reporting on recurrent pharyngeal abscesses and their management. Results were limited to articles published in English from inception to August 2021. The timely management of pharyngeal infections acutely obstructing the airway is crucial. Physicians should adopt close and frequent monitoring and have a low threshold for reimaging should symptoms worsen or fail to improve after the initial surgical intervention.

## Introduction

Pharyngeal abscesses are distinctive among infectious processes given their propensity to develop severe, life-threatening complications.^1^ Early detection and treatment of such deep neck infections are critical, but often complicated by the intricate head and neck anatomy.^1^ In the postantibiotic era, clinicians have seen a dramatic decline in the incidence of pharyngeal infection.^2^ Regardless, otolaryngologists continue to routinely observe and manage abscesses in the peritonsillar, parapharyngeal, and retropharyngeal spaces.

Airway obstruction due to abscesses in the oropharyngeal and hypopharyngeal spaces is very scarcely reported, and thus, evidence-based guidelines for contemporary management are lacking. Generally, surgical drainage is considered the mainstay of treatment, but recent studies have proposed conservative management with antibiotics for cases of uncomplicated deep neck abscesses.^1^ Management guidelines for recurrent deep neck abscesses are lacking further still and may reflect the complex etiology and rarity of such cases. The current literature has proposed predisposing factors to recurrent deep neck infection, such as diabetes^3^ and smoking behavior,^4^ but the underlying etiology remains unclear.

Herein, we describe the case of an 83-year-old male that presented with an obstructive, infratonsillar oropharyngeal abscess extending to the hypopharynx, which recurred after initial surgical management 12 years prior. We also review the current literature on etiology and management of recurrent pharyngeal abscesses. The following case was reported in accordance with the CARE case report reporting guidelines.

## Case report

An 83-year-old man presented to the emergency department in April 2020 with 8 days of left-sided neck pain, sore throat, dysphagia, odynophagia, dysphonia, and worsening of his baseline dyspnea. Past medical history was pertinent for poorly controlled type 2 diabetes, obstructive sleep apnea, a pacemaker for symptomatic bradycardia, and severe coronary artery disease requiring percutaneous coronary intervention for nine stents. He had a 40-pack-year history of smoking but had been an ex-smoker for 22 years.

**Figure 1.**
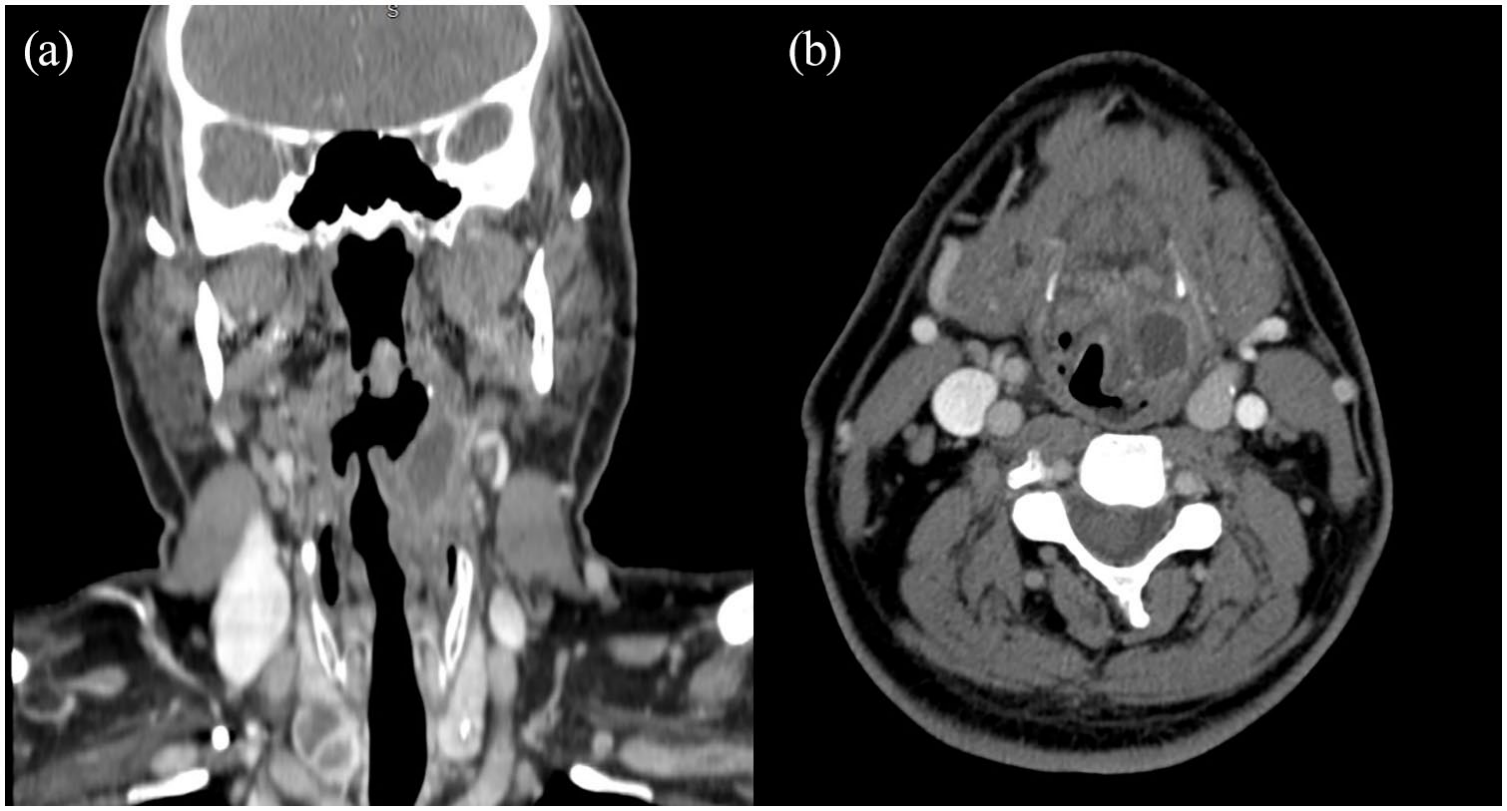
CT with contrast of the head and neck showing a large left laryngeal mass (2008): (a) coronal view and (b) axial view.

He reported a history of a similar constellation of upper airway obstructive symptoms in 2008 that resulted from an oropharyngeal abscess. On presentation in 2008, an oropharyngeal abscess causing airway compromise (Figure 1) required urgent direct laryngoscopy and incision of the abscess with a sickle knife. Cultures of the pus revealed enterobacter and a mild amount of candida. He was started on a course of oral trimethoprim and sulfamethoxazole and fluconazole. Following the initial surgical intervention, the patient showed some clinical improvement but daily flexible nasolaryngoscopy and a repeat computed tomography (CT) showed persistence of the abscess. On postoperative day 5, he was taken back to the operating theater for a second incision and drainage. The patient was reviewed 1 week later in the clinic and had complete normalization of the larynx on flexible nasolaryngoscopy.

**Figure 2.**
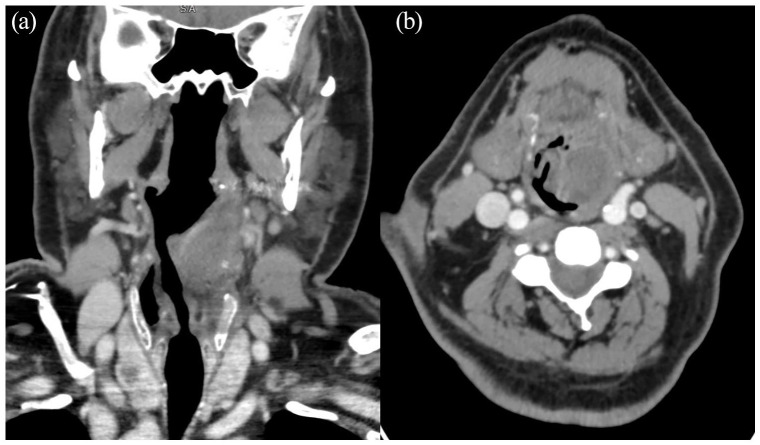
CT with contrast of the head and neck showing a large left laryngeal mass (2020): (a) coronal view and (b) axial view.

Upon arrival to the emergency department in April 2020, the patient was dyspneic, but able to manage his secretions. On examination, the oral cavity appeared within normal limits. Tonsils were symmetrical (2+) without tonsillar/peritonsillar edema, exudate, or erythema. Flexible nasolaryngoscopy revealed a large oropharyngeal mass with normal overlying mucosa in the same location as his 2008 abscess. The mass displaced the epiglottis to the right, obstructing 80%–90% of the airway. The epiglottis and base of the tongue appeared within normal limits. A complete blood count was significant for mild leukocytosis (11 × 10^9^/L). CT with contrast confirmed a 3.0 × 2.2 × 2.9 cm oropharyngeal abscess effacing the left vallecula and causing a mass effect on the aryepiglottic folds (Figure 2).

The patient was taken to the operative theater for direct laryngoscopy and awake orotracheal fibreoptic intubation was achieved. The mass was opened extensively with endoscopic scissors and pus was expressed. Cultures and biopsies were obtained. The patient was continued on intravenous Cefazolin and Flagyl antibiotics and intravenous dexamethasone. The patient was extubated the following morning without incident.

On postoperative day 2, the patient developed progressive dysphonia and dysphagia, similar to his initial presentation. A repeat CT with contrast revealed reaccumulation of the collection, causing significant (>75%) airway reobstruction. This time, a combination of endoscopic scissors and a laryngeal microdebrider was used to partially resect the abscess wall to widely open the collection until it was flush with the pharyngeal wall. Intravenous antibiotics were broadened to piperacillin/tazobactam. Postoperatively, cultures showed no growth and pathology revealed no abnormalities. After full recovery, the patient was discharged on postoperative day 4. Repeat flexible nasolaryngoscopy in August 2020 demonstrated complete normalization of the pharynx.

## Discussion

In this case, we report an unusual presentation of a recurrent, obstructive, oropharyngeal abscess on two separate occasions. The etiology of the recurrent infections remains unclear.

We performed a detailed search of the literature for studies reporting on recurrent pharyngeal abscesses and their management. Electronic databases (MEDLINE) were searched with a trained librarian from inception until 23 August 2021, without language restrictions. The following keywords and MeSH terms were used in varying combinations: recurrent, relapse, reinfection, oropharyngeal, parapharyngeal, infratonsillar, abscess, infection, and cyst. The search identified a total of 585 potential studies. We excluded non-English articles from title and abstract screening and further excluded studies based on relevance to our search. References of systematic reviews and key articles were screened for potentially relevant articles. A total of seven studies were included in the qualitative synthesis.^5–11^ The studies were published from 1997 to 2020. The studies were published from 1997 to 2020. Recurrent oropharyngeal abscesses were predominantly reported in age 18–65 years. All abscesses were investigated with CT and required surgical drainage for management.

The recurrence of an identical abscess 12 years after initial drainage suggests a possible underlying unascertained etiology. Retention cysts are the most common benign lesions of the pharynx, albeit rare. In a recent literature review^12^ of hypopharyngeal retention cysts, the authors noted that these cases are likely underestimated as 50% of the lesions are not easily visible on endoscopy due to their submucosal origin and normal overlying mucosa. In contrast to this case presentation, it is suggested that these pharyngeal cysts rarely become secondarily infected and that recurrence after surgical management is uncommon.^12^ Abscesses originating in the hypopharyngeal wall are extremely scarce and unlike this case, and are typically secondary to a foreign body,^13–15^ phlegmonous esophagogastritis,^16,17^ or as a complication of cervical osteophytes.^18^ Likewise, a pharyngeal infection may be an initial presenting symptom of hypopharyngeal cancer, due to necrotic lymph nodes and subsequent abscess formation.^19^ Branchial anomalies can lead to pharyngeal and deep neck infections. Third and fourth branchial pouch abnormalities, arising from the pyriform sinuses and extending to the upper thyroid lobes^20^ have the lowest incidence.^21–23^ Incomplete obliteration of these pouches can lead to cysts, sinuses, and fistulas, or may present as pharyngeal infections in adults.^11,20,22–24^ Interestingly, and in parallel with our case, the majority of these sinus tracts are present on the left side.^25–27^ However, there was no history or evidence of branchial anomalies identified in this case.

Interestingly, the recurrence time reported in the literature is typically less than 1 year.^5,6^ The 12-year recurrence time observed in the case herein suggests a complex constellation of contributing factors, as outlined previously, and underscores the unique presentation of this case.

Contextualization of our case regarding recurrence is challenging, as the affected anatomical space is unique in the literature. The reported case was localized to the infratonsillar region and extended down to the level of the hypopharynx, which differs from the more commonly observed peritonsillar abscess. Peritonsillar abscesses, the most common deep neck space infections, refer specifically to infection of the Weber glands, which results in a nidus of pus accumulation between the capsule of the palatine tonsils and the superior constrictor muscles of the pharynx localized superior to the tonsil.^28–31^ However, there are rare reports of peritonsillar abscesses of the inferior pole in the literature, which should be considered in the differential diagnosis for this case.^32^ In addition, isolated cases of atypical oropharygneal abscesses have been reported previously in the literature: infratonsillar abscesses resultant from infection of the lingual tonsils,^33–36^ palatine tonsils^37–41^ as well as peritonsillar abscesses posttonsillectomy.^42^ However, the majority of these patients were pediatric and none of them presented with severe life-threatening airway obstruction due to their localization in the peritonsillar region. Noninfectious, congenital oropharyngeal cysts have been reported in the literature that have been large enough to cause dysphagia^36^ and intermittent airway obstruction.^37^ However, these have been limited to the pediatric population.

An immunocompromised state secondary to concomitant type 2 diabetes might have contributed to the occurrence and recurrence of the abscess in our patient. It has been shown that patients presenting with deep neck infections have over a threefold incidence of diabetes when compared to the rest of the population,^3^ suggesting that it might be playing a role in the underlying pathophysiology. In line with this, diabetes-induced immunosuppression decreases the antibacterial neutrophil function,^43^ easing infection and abscess formation. Furthermore, smoking has been shown to alter oral mucosa and microbiome, predisposing individuals to peritonsillar abscess formation.^4^ Given the association between smoking behavior and peritonsillar abscess,^44^ our patient’s smoking history may have also contributed to the recurrence of the abscess.

Oropharyngeal abscesses are traditionally managed with a transoral approach but optimal techniques and postoperative management, particularly for recurrent abscesses, are lacking.^45^ Established treatments are only available for recurrent abscesses that arise from congenital anomalies, such as branchial cleft anomalies^7–9^ and pyriform sinus fistulas.^10,11^ For these cases, surgical management of the underlying congenital anomaly successfully prevents further abscess recurrence. Furthermore, while tonsillectomy might seem like a reasonable approach to mitigate recurrence, evidence suggests that adult patients having received the surgery are at a higher risk of pharyngeal infections.^46^ Likewise, a nation-wide epidemiological study has found an inverse relationship with the decline in elective tonsillectomy and pharyngeal infections.^47^

The case presented here highlights the need for surgeons to exercise vigilance in monitoring patients with transoral approaches to oropharyngeal abscesses postoperatively, given their increased propensity to reaccumulate rapidly. The widely accepted treatment algorithm for abscesses that are drained with a cutaneous incision, that is, incision, drainage, and packing the wound with gauze to permit healing to take place by secondary intention or placement of a subcutaneous drain to reduce reaccumulation of the infectious material,^48–52^ cannot be applied to oropharyngeal abscesses. The rationale behind wound packing revolves around placing enough material to keep the walls of the abscess separated, allowing further drainage, and preventing the formation of a subsequent pyogenic pocket. Without the ability to place packing or a drain to maintain the patency of the abscess, extensive incision, and drainage is needed to reduce reaccumulation. Indeed, the current literature highlights incision and drainage with antibiotic therapy to be useful in the management of recurrent abscesses. The antibiotic therapy, however, lacks consistency between studies as there is currently no established guideline to inform antibiotic selection for recurrent abscesses.^5,6^

In the case presented herein, wide incision and drainage of the oropharyngeal abscess, with partial resection of the overlying oropharyngeal mucosa, was required for definitive management. This technique allowed the wound to continually drain and prevents its premature closure and subsequent reaccumulation. The use of a laser can also be considered as some surgeons have found it to be useful at creating a wide opening to incise pharyngeal abscesses.^53^

## Conclusion

This case presents a patient with a recurring obstructive abscess of the oropharynx. The timely management of pharyngeal infections acutely obstructing the airway is crucial. Special consideration must be given to diabetic patients, as their capacity to fight the infection might be compromised. Furthermore, the identical recurrence of a lesion should prompt further imaging and work-up for congenital etiologies, as branchial anomalies might be the underlying cause. A review of current literature reveals a paucity of evidence on recurrent abscesses and their management. However, prompt extensive drainage of the abscess followed by antibiotic therapy is suggested to prevent rapid reaccumulation. Finally, physicians should adopt close and frequent monitoring and have a low threshold for reimaging should symptoms worsen or fail to improve after the initial surgical intervention.

## References

[1] Vieira F Allen SM Stocks RMS , et al. Deep neck infection. Otolaryngol Clin North Am 2008; 41(3): 459–483, vii. 18435993 10.1016/j.otc.2008.01.002

[2] Chow AW . Life-threatening infections of the head, neck, and upper respiratory tract. In: Hall JB Schmidt GA Kress JP (eds) Principles of critical care. 4th ed. New York: McGraw-Hill Education, 2015, accessmedicine.mhmedical.com/content.aspx?aid=1107721092 (accessed 7 May 2020).

[3] Buckley J Harris AS Addams-Williams J . Ten years of deep neck space abscesses. J Laryngol Otol 2019; 133(4): 324–328. 30924432 10.1017/S0022215119000458

[4] Lehnerdt G Senska K Fischer M , et al. Rauchen prädisponiert zum Peritonsillarabszess [Smoking promotes the formation of peritonsillar abscesses]. Laryngorhinootologie 2005; 84(9): 676–679. 16142623 10.1055/s-2005-870289

[5] Wikstén JE Pitkäranta A Blomgren K . Metronidazole in conjunction with penicillin neither prevents recurrence nor enhances recovery from peritonsillar abscess when compared with penicillin alone: a prospective, double-blind, randomized, placebo-controlled trial. J Antimicrob Chemother 2016; 71(6): 1681–1687. 26968881 10.1093/jac/dkw038

[6] Tokui N . Recurrent deep neck abscess caused by Enterobacter cloacae in the elderly. Otolaryngol Head Neck Surg 2005; 77(8): 585–588.

[7] Ning Y Li C Wang X , et al. Resection of second, third, and fourth branchial cleft anomalies with recurrent or repeated neck infection using the selective neck dissection technique. ORL J Otorhinolaryngol Relat Spec 2020; 82(2): 59–66. 32092758 10.1159/000501893

[8] Burstin PP Briggs RJ . Fourth branchial sinus causing recurrent cervical abscess. Aust N Z J Surg 1997; 67(2–3): 119–122. 9068553 10.1111/j.1445-2197.1997.tb01915.x

[9] Daher P Francis E Raffoul L , et al. Ectopic gastric mucosa in the cervical esophagus presenting as a recurrent neck abscess: a case report. J Pediatr Surg 2010; 45(6): e15–e17. 10.1016/j.jpedsurg.2010.03.022 20620294

[10] Laababsi R Elbouhmadi K Bouzbouz A , et al. Misdiagnosed pyriform sinus fistula revealed by iterative neck abscesses: a case report and review of the literature. Ann Med Surg 2020; 59: 64–67. 10.1016/j.amsu.2020.08.051 PMC7501404 32994985

[11] Zhang P Tian X . Recurrent neck lesions secondary to pyriform sinus fistula. Eur Arch Otorhinolaryngol 2016; 273(3): 735–739. 25708412 10.1007/s00405-015-3572-2

[12] Ahmed ME Ahmed ME-R El Batawi AM , et al. Internal hypopharyngeal cyst: a review of literature. Dysphagia 2019; 34(4): 487–498. 30927081 10.1007/s00455-019-10003-2

[13] Richter K . (Submucosal foreign body (sewing needle) in the wall of the hypopharynx; abscess formation, incision and extraction through the pharynx). HNO 1955; 5(3): 91. 13262631

[14] Heyworth P Shulman R . A Christmas message: be careful of the confetti stars. Med J Aust 2019; 211(11): 510. 31737906 10.5694/mja2.50424

[15] Mowinckel MS Charabi BW . (Migrating foreign body from hypopharynx). Ugeskr Laeg 2014; 176(37): V04140250. 25294034

[16] Huang Y-C Cheng C-Y Liao C-Y , et al. A rare case of acute phlegmonous esophagogastritis complicated with hypopharyngeal abscess and esophageal perforation. Am J Case Rep 2017; 18: 125–130. 28163299 10.12659/AJCR.902180 PMC5308544

[17] Shiozawa K Watanabe M Ikoma A , et al. (Case of phlegmonous esophagogastritis associated with hypopharyngeal abscess). Nihon Shokakibyo Gakkai Zasshi 2009; 106(3): 370–376. 19262050

[18] Angelos C Dimitra A . Dysphagia due to anterior cervical osteophytes complicated with hypopharynx abscess. BMJ Case Rep 2011; 2011: bcr1120103551, https://www.ncbi.nlm.nih.gov/pmc/articles/PMC3062881/ (accessed 29 April 2020). 10.1136/bcr.11.2010.3551 PMC3062881 22707552

[19] Ridder GJ Eglinger CF Technau-Ihling K , et al. Deep neck abscess masquerading hypopharyngeal carcinoma. Otolaryngol Head Neck Surg 2000; 123(5): 659–660. 11077367 10.1067/mhn.2000.110613

[20] Lachance S Chadha NK . Systematic review of endoscopic obliteration techniques for managing congenital piriform fossa sinus tracts in children. Otolaryngol Head Neck Surg 2016; 154(2): 241–246. 26527612 10.1177/0194599815613286

[21] Choi SS Zalzal GH . Branchial anomalies: a review of 52 cases. Laryngoscope 1995; 105(9 Pt 1): 909–913. 7666723 10.1288/00005537-199509000-00007

[22] Ford GR Balakrishnan A Evans JN , et al. Branchial cleft and pouch anomalies. J Laryngol Otol 1992; 106(2): 137–143. 1556487 10.1017/s0022215100118900

[23] Adamidou F Anagnostis P Karras S , et al. Neck abscess associated with a piriform fossa sinus tract in an adult. BMJ Case Rep 2013; 2013: bcr2013010119. 10.1136/bcr-2013-010119 PMC3703053 23780773

[24] Pal I Kumar S Mukherjee A , et al. Fourth branchial pouch sinus: a report of 7 cases and review of the literature. Ear Nose Throat J 2018; 97(8): 236–242. 30138515 10.1177/014556131809700820

[25] Yang C Cohen J Everts E , et al. Fourth branchial arch sinus: clinical presentation, diagnostic workup, and surgical treatment. Laryngoscope 1999; 109(3): 442–446. 10089973 10.1097/00005537-199903000-00019

[26] Jordan JA Graves JE Manning SC , et al. Endoscopic cauterization for treatment of fourth branchial cleft sinuses. Arch Otolaryngol Head Neck Surg 1998; 124(9): 1021–1024. 9738814 10.1001/archotol.124.9.1021

[27] Garrel R Jouzdani E Gardiner Q , et al. Fourth branchial pouch sinus: from diagnosis to treatment. Otolaryngol Head Neck Surg 2006; 134(1): 157–163. 16399198 10.1016/j.otohns.2005.05.653

[28] Klug TE Rusan M Fuursted K , et al. Peritonsillar abscess: complication of acute tonsillitis or Weber’s glands infection? Otolaryngol Head Neck Surg 2016; 155(2): 199–207. 27026737 10.1177/0194599816639551

[29] Sowerby LJ Hussain Z Husein M . The epidemiology, antibiotic resistance and post-discharge course of peritonsillar abscesses in London, Ontario. J Otolaryngol Head Neck Surg 2013; 42: 5. 23663820 10.1186/1916-0216-42-5 PMC3646551

[30] Herzon F Harris P . Mosher award thesis. Peritonsillar abscess: incidence, current management practices, and a proposal for treatment guidelines. Laryngoscope 1995; 105(8 Pt 3, Suppl. 74): 1–17. 10.1288/00005537-199508002-00001 7630308

[31] Risberg S Engfeldt P Hugosson S . Incidence of peritonsillar abscess and relationship to age and gender: retrospective study. Scand J Infect Dis 2008; 40(10): 792–796. 18609198 10.1080/00365540802195226

[32] Licameli GR Grillone GA . Inferior pole peritonsillar abscess. Otolaryngol Head Neck Surg 1998; 118(1): 95–99. 9450835 10.1016/S0194-5998(98)70381-X

[33] Awai S Miller BJ Dimitrov L , et al. Lingual tonsil abscess: a rare, life-threatening cause of acute sore throat. BMJ Case Rep 2019; 12(5): e229555. 10.1136/bcr-2019-229555 PMC6536224 31126932

[34] Coughlin AM Baugh RF Pine HS . Lingual tonsil abscess with parapharyngeal extension: a case report. Ear Nose Throat J 2014; 93(9): E7–E8. 10.1177/014556131409300902 25255362

[35] Srivanitchapoom C Yata K . Lingual abscess: predisposing factors, pathophysiology, clinical manifestations, diagnosis, and management. Int J Otolaryngol 2018; 2018: 4504270. 30524479 10.1155/2018/4504270 PMC6247437

[36] McMullen CP Frank DK Smith LP . Backyard hazard: a case series of ingested grill brush bristles and a novel approach to extraction. Am J Otolaryngol 2012; 33(6): 731–734. 22917952 10.1016/j.amjoto.2012.07.004

[37] Giurintano JP Kortebein S Sebelik M , et al. Intratonsillar abscess: a not-so-rare clinical entity. Int J Pediatr Otorhinolaryngol 2019; 119: 38–40. 30665174 10.1016/j.ijporl.2018.12.039

[38] Wang AS Stater BJ Kacker A . Intratonsillar abscess: 3 case reports and a review of the literature. Int J Pediatr Otorhinolaryngol 2013; 77(4): 605–607. 23375749 10.1016/j.ijporl.2012.12.034

[39] Ali SA Kovatch KJ Smith J , et al. Predictors of intratonsillar versus peritonsillar abscess: a case-control series. Laryngoscope 2019; 129(6): 1354–1359. 30569506 10.1002/lary.27615 PMC6755033

[40] Añaguari BN Rebollo J Montes C . Intratonsillar abscess, a rare cause of odynophagia. Acta Otorrinolaringol Esp (Engl Ed) 2017; 68(4): 246–247. 27562902 10.1016/j.otorri.2016.05.004

[41] Ahmed Ali S Kovatch KJ Smith J , et al. Predictors of intratonsillar abscess versus peritonsillar abscess in the pediatric patient. Int J Pediatr Otorhinolaryngol 2018; 114: 143–146. 30262353 10.1016/j.ijporl.2018.08.042

[42] Stankiewicz JA Talland C . Peritonsillarlike lateral oropharyngeal abscess after tonsillectomy. Arch Otolaryngol Head Neck Surg 1988; 114(10): 1181–1183. 3415828 10.1001/archotol.1988.01860220115036

[43] Delamaire M Maugendre D Moreno M , et al. Impaired leucocyte functions in diabetic patients. Diabet Med 1997; 14(1): 29–34. 9017350 10.1002/(SICI)1096-9136(199701)14:1<29::AID-DIA300>3.0.CO;2-V

[44] Schwarz D Wolber P Balk M , et al. Analysis of smoking behaviour in patients with peritonsillar abscess: a prospective, matched case-control study. J Laryngol Otol 2018; 132(10): 872–874. 30208977 10.1017/S0022215118001585

[45] Tsai T-Y Su C-Y . Surgical technique of transoral marsupialization for the treatment of nasopharyngeal branchial cysts. Ann Otol Rhinol Laryngol 2010; 119(5): 336–341. 20524580 10.1177/000348941011900511

[46] Kim SY Min C Lee WH , et al. Tonsillectomy increases the risk of retropharyngeal and parapharyngeal abscesses in adults, but not in children: a national cohort study. PLoS ONE 2018; 13(3): e0193913, https://www.ncbi.nlm.nih.gov/pmc/articles/PMC5839582/ (accessed 20 May 2020). 10.1371/journal.pone.0193913 PMC5839582 29509810

[47] Windfuhr JP Chen Y-S . Hospital admissions for acute throat and deep neck infections versus tonsillectomy rates in Germany. Eur Arch Otorhinolaryngol 2019; 276(9): 2519–2530. 31214826 10.1007/s00405-019-05509-2

[48] Prabhu SR Nirmalkumar ES . Acute fascial space infections of the neck: 1034 cases in 17 years follow up. Ann Maxillofac Surg 2019; 9(1): 118–123. 31293939 10.4103/ams.ams_251_18 PMC6585228

[49] Ruth H Davis WE Renner G . Deep neck abscess after tracheoesophageal puncture and insertion of a voice button prosthesis. Otolaryngol Head Neck Surg 1985; 93(6): 809–811. 3937107 10.1177/019459988509300622

[50] Takao M Ido M Hamaguchi K , et al. Descending necrotizing mediastinitis secondary to a retropharyngeal abscess. Eur Respir J 1994; 7(9): 1716–1718. 7995405 10.1183/09031936.94.07091716

[51] Langenbrunner DJ Dajani S . Pharyngomaxillary space abscess with carotid artery erosion. Arch Otolaryngol 1971; 94(5): 447–457. 5114954 10.1001/archotol.1971.00770070693011

[52] Makihara S Kariya S Naito T , et al. False vocal cord perforation with abscess treated by negative pressure wound therapy. SAGE Open Med Case Rep 2020; 8: 2050313X20915415, https://www.ncbi.nlm.nih.gov/pmc/articles/PMC7139174/ (accessed 16 May 2020). 10.1177/2050313X20915415 PMC7139174 32284867

[53] Nicolai P Lombardi D Berlucchi M , et al. Drainage of retro-parapharyngeal abscess: an additional indication for endoscopic sinus surgery. Eur Arch Otorhinolaryngol 2005; 262(9): 722–730. 15668811 10.1007/s00405-004-0890-1

